# Fatty acid binding protein type 7 deficiency preserves auditory function in noise-exposed mice

**DOI:** 10.1038/s41598-023-48702-4

**Published:** 2023-12-06

**Authors:** Jun Suzuki, Tomotaka Hemmi, Masamitsu Maekawa, Masahiro Watanabe, Hitoshi Inada, Hiroyuki Ikushima, Tetsuya Oishi, Ryoukichi Ikeda, Yohei Honkura, Yoshiteru Kagawa, Tetsuaki Kawase, Nariyasu Mano, Yuji Owada, Noriko Osumi, Yukio Katori

**Affiliations:** 1 Department of Otolaryngology-Head and Neck Surgery, Tohoku University Graduate School of Medicine, 1-1 Seiryo-machi, Aoba-ku, Sendai, Miyagi 980-8574 Japan; 2 Department of Pharmaceutical Sciences, Tohoku University Hospital, 1-1 Seiryo-machi, Aoba-ku, Sendai, Miyagi 980-8574 Japan; 3 Graduate School of Pharmaceutical Sciences, Tohoku University, 1-1 Seiryo-machi, Aoba-ku, Sendai, Miyagi 980-8574 Japan; 4 Department of Developmental Neuroscience, Centers for Neuroscience, Tohoku University Graduate School of Medicine, 2-1 Seiryo-machi, Aoba-ku, Sendai, Miyagi 980-8575 Japan; 5 Department of Otolaryngology, Head and Neck Surgery, Iwate Medical University School of Medicine, 19-1 Odori, Yahaba, Shiwa, 020-8505 Japan; 6 Department of Organ Anatomy, Tohoku University Graduate School of Medicine, 2-1 Seiryo-machi, Aoba-ku, Sendai, Miyagi 980-8575 Japan

**Keywords:** Cochlea, Hair cell

## Abstract

Fatty acid-binding protein 7 (FABP7) is vital for uptake and trafficking of fatty acids in the nervous system. To investigate the involvement of FABP7 in noise-induced hearing loss (NIHL) pathogenesis, we used *Fabp7* knockout (KO) mice generated via CRISPR/Cas9 in the C57BL/6 background. Initial auditory brainstem response (ABR) measurements were conducted at 9 weeks, followed by noise exposure at 10 weeks. Subsequent ABRs were performed 24 h later, with final measurements at 12 weeks. Inner ears were harvested 24 h after noise exposure for RNA sequencing and metabolic analyses. We found no significant differences in initial ABR measurements, but *Fabp7* KO mice showed significantly lower thresholds in the final ABR measurements. Hair cell survival was also enhanced in *Fabp7* KO mice. RNA sequencing revealed that genes associated with the electron transport chain were upregulated or less impaired in *Fabp7* KO mice. Metabolomic analysis revealed various alterations, including decreased glutamate and aspartate in *Fabp7* KO mice. In conclusion, FABP7 deficiency mitigates cochlear damage following noise exposure. This protective effect was supported by the changes in gene expression of the electron transport chain, and in several metabolites, including excitotoxic neurotransmitters. Our study highlights the potential therapeutic significance of targeting FABP7 in NIHL.

## Introduction

Noise-induced hearing loss (NIHL) is a sensorineural hearing impairment caused by acute, single, chronic, or repeated exposure to loud sounds. While chronic occupational noise exposure is an important cause of NIHL, nonoccupational noise from residential and recreational activities has recently attracted attention as a contributing factor to NIHL^1–3^. According to a recent systematic review, more than 1 billion young people are at risk of hearing loss due to unsafe listening practices such as using personal listening devices and attending loud entertainment venues^4^. A recent study reported the deterioration of hearing thresholds at 1000, 2000, and 4000 Hz among the younger generation during the later decade (2010–2020) compared to those in the former decade (2000–2010). This suggests that NIHL may explain the observed changes^5^. Preventative measures, such as reducing noise volume, limiting exposure time, and wearing hearing protection, are crucial in mitigating the risk of NIHL^2^. However, despite their importance, these preventive methods are not widely practiced. Hence, there is an increasing demand for prophylactic and therapeutic drugs for NIHL^6^, since no established medication is available for humans.

The pathogenesis of NIHL is complex and involves both genetic and environmental factors^2,7,8^. Although various factors, such as oxidative stress, inflammation, and glutamate excitotoxicity, have been proposed as crucial contributors to NIHL development in mammals, the underlying molecular mechanisms are not yet fully understood^2,7,9,10^. The excessive generation of reactive oxygen species (ROS) is widely acknowledged as a mediator of noise-induced cochlear damage, and ROS-induced lipid peroxidation products can induce apoptosis, resulting in the permanent loss of auditory sensory cells in the cochlea^7^. Polyunsaturated fatty acids (PUFAs), including docosahexaenoic acid (DHA) and arachidonic acid (ARA), affect many physiological processes and are particularly susceptible to oxidation because of their high degrees of unsaturation, and their oxidized products can exert toxic effects^11^. Previous studies found that genetic enrichment or dietary supplementation with *n*-3 PUFA slows the progression of age-related hearing loss (AHL) in mice^12,13^. Moreover, the intake of *n*-3 PUFAs or the consumption of fish is inversely correlated with AHL progression^14^, and an inverse correlation exists between plasma *n*-3 PUFA levels and AHL progression in humans^15^. However, a perinatal diet supplemented with high levels of DHA or *n*-3 PUFAs may harm the auditory system in rats^16,17^. Therefore, the precise role of PUFAs in hearing remains inconclusive^12–20^. To our knowledge, no previous studies have investigated the relationship between the pathology of NIHL and PUFAs.

PUFAs are hydrophobic molecules that bind to fatty acid-binding proteins (FABPs) in the aqueous cytoplasm. FABPs play crucial roles in facilitating the cellular uptake and intracellular trafficking of fatty acids, thereby regulating metabolic pathways and gene expression^21–23^. Among the FABP family, FABP7 exhibits preferential binding affinity to *n*-3 PUFAs, particularly DHA, and contributes to cell membrane production and synaptic vesicles^22^. FABP7 also attracts attention as a regulator of cell signaling in tumors such as glioma, melanoma, and renal cancer^24^. The expressions of FABP3 and FABP7 differ significantly in the mouse cochlea. FABP3 is specifically expressed in spiral ganglion (SG) neurons and supporting cells within the organ of Corti (OC), whereas FABP7 is predominantly found in non-hair cells and non-neuronal cells, including supporting cells in the OC, fibrocytes in the spiral limbus (SLim) and spiral ligament (SLig), and satellite cells in the SG^25–27^. *Fabp3* deficiency does not affect auditory function^27^. Our previous study involving *Fabp7* knockout (KO) C57BL/6 mice, generated using embryonic stem cells of the 129/Sv background, suggested a delay in the progression of age-related hearing loss in these mice^26^. However, subsequent findings revealed that these *Fabp7* KO mice possess a normal *Cdh23*^753^^G^ allele derived from the 129/Sv strain, raising uncertainty as to whether *Fabp7* deficiency alone is responsible for the observed hearing-protective phenotype^26^. Therefore, this study aimed to investigate the effects of *Fabp7* deficiency on hearing following noise exposure by evaluating *Fabp7*-KO mice on a C57BL/6 background, established via the CRISPR-Cas9 system^16,28^.

## Results

### Fabp7 KO mice showed normal growth in standard environmental conditions

**Figure 1:**
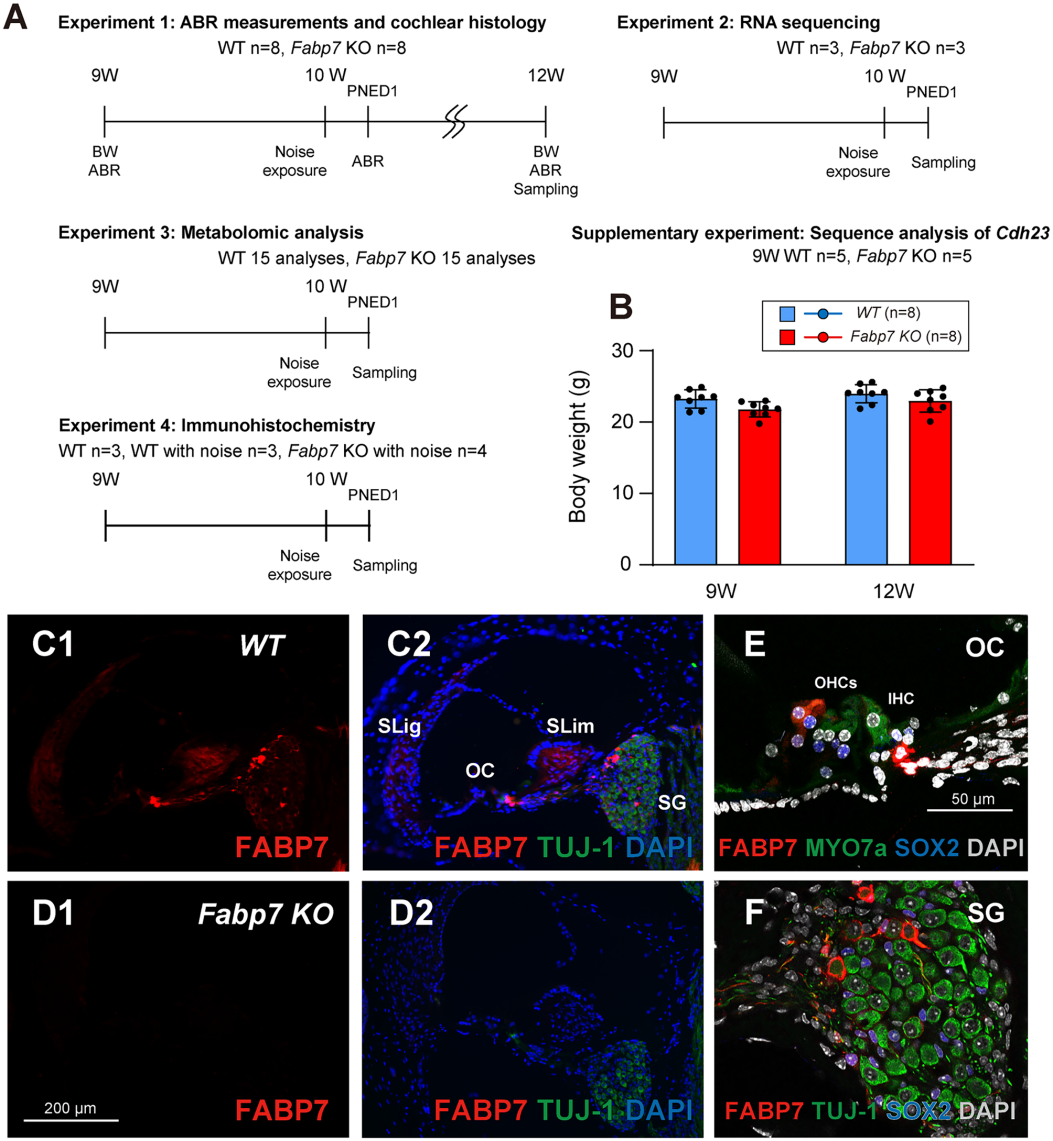
Experimental design, body weights, and FABP7 expression in the cochlea. Schematic of the experiments using wild-type (WT) and *Fabp7* knockout (KO) mice (**A**). The body weight (BW) of *Fabp7* KO mice was not significantly different from that of WT mice (**B**). Immunohistochemistry of FABP7 in the cochlea of WT mice revealed that FABP7 was expressed throughout the spiral ganglion (SG), organ of Corti (OC), spiral limbus (SLim), and spiral ligament (SLig) (**C**). Scale bar, 200 μm (**C**–**D**). No expression of FABP7 was observed in the cochlea of *Fabp7* KO mice (**D**). Maximum projection images of the OC showing high expression of FABP7 in Schwann cells and SOX2-positive supporting cells around the MYO7a-positive outer hair cells (OHCs) in the cochlea of WT mice (**E**). In the SG, FABP7 was expressed in satellite cells surrounding TUJ-1-positive neurons (**F**). Scale bars, 50 μm (**E**–**F**). Auditory brainstem response; ABR, weeks; W, post-noise exposure day; PNED, inner hair cell; IHC. Statistical significance was determined using two-way analysis of variance, followed by Šídák multiple comparison test. Error bars represent standard deviation.

Wild-type (WT) (n = 8) and *Fabp7* KO mice (n = 8) were weighed at 9 and 12 weeks of age, to evaluate the effects of *Fabp7* deletion on body weight (Fig. 1A). There were no significant differences in body weights between the two groups at any time point during the experiment (Fig. 1B). These results indicate that *Fabp7* deletion has no effect on body weight in the youth during growth stages.

To confirm that the newly designed *Fabp7* KO mice had a single nucleotide polymorphism in the *Cdh23* gene (*Cdh23*^753^^A^) on chromosome 10, which is the most critical genetic variant causing hearing loss in the C57BL/6 strain^26^, we performed sequence analysis of 5 mice per group (Fig. 1A). All the WT and *Fabp7* KO mice examined had the *Cdh*23^753A^ genotype (Supplementary Fig. 1), suggesting that the mice examined in this study had a WT genetic background of *Cdh23*.

Next, we assessed FABP7 expression in the cochleae of WT and *Fabp7* KO mice by immunohistochemistry of frozen sections to investigate whether FABP7 expression was completely absent in the cochlea of *Fabp7* KO mice (Fig. 1A). As expected, we confirmed the presence of FABP7 in various cell types, such as Schwann cells and supporting cells in the OC, satellite cells in the SG, fibrocytes in the SLim and SLig, in the cochlea of WT mice in a similar pattern to previous reports^25,26^ (Fig. 1C,E,F). Furthermore, we confirmed the complete loss of FABP7 expression in the cochlea of *Fabp7* KO mice established via the CRISPR-Cas9 system (Fig. 1D).

### Fabp7 deletion protects from hearing deterioration after noise exposure

**Figure 2:**
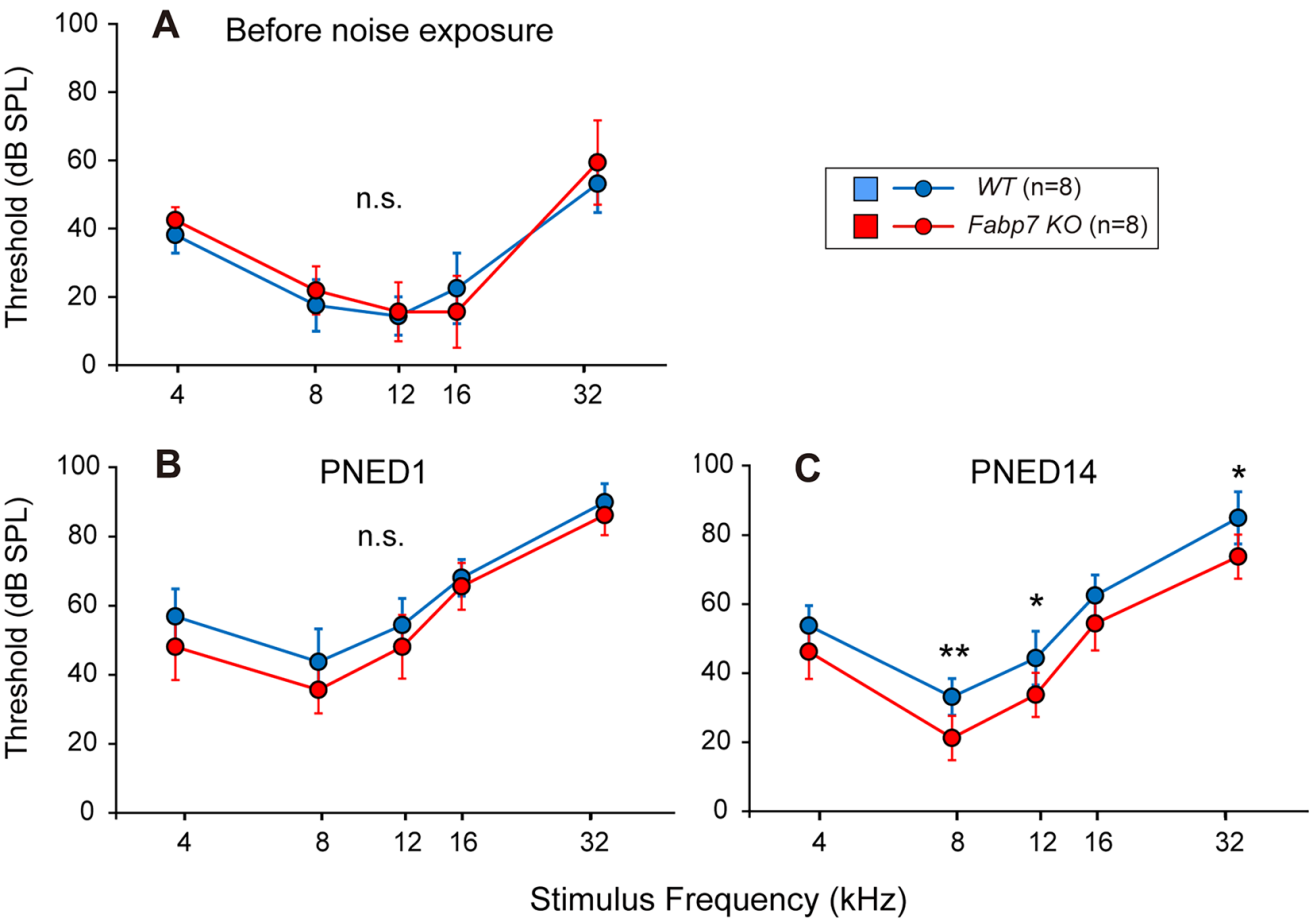
ABR thresholds before and after noise exposure. No significant differences between wild-type (WT) and *Fabp7* knockout (KO) mice in the auditory brainstem response (ABR) thresholds before noise exposure (**A**) and at post-noise exposure day (PNED) 1 (**B**). The ABR thresholds of *Fabp7* KO mice at 8, 12, and 32 kHz were significantly lower than those of WT mice at PNED 14 (**C**). Statistical significance was determined using two-way analysis of variance, followed by Šídák multiple comparison test. n.s., not significant; *P < 0.05; **P < 0.01. Error bars represent standard deviation.

To address whether *Fabp7* deficiency affects susceptibility to noise exposure causing moderate permanent threshold shift (PTS, approximately 50 dB sound pressure level (SPL)), auditory brainstem response (ABR) thresholds of WT mice (n = 8) and *Fabp7* KO mice (n = 8) were examined before and after exposure to 8–16 kHz octave-band noise at 100 dB SPL for 2 h (Fig. 1A). The ABR thresholds before and at post-noise exposure day (PNED) 1 were not significantly different between the WT and *Fabp7* KO mice (Fig. 2A,B), indicating that FABP7 is not crucial for normal development and function of the cochlea and protection from initial cochlear damage after noise exposure. However, the ABR thresholds at 8, 12, and 32 kHz in *Fabp7* KO mice at PNED 14 were significantly lower than those in WT mice (Fig. 2C). These results suggested that *Fabp7* deletion protected against permanent hearing deterioration after exposure to noise causing moderate PTS in mice.

### Fabp7 deletion protects loss of hair cells after noise exposure

**Figure 3:**
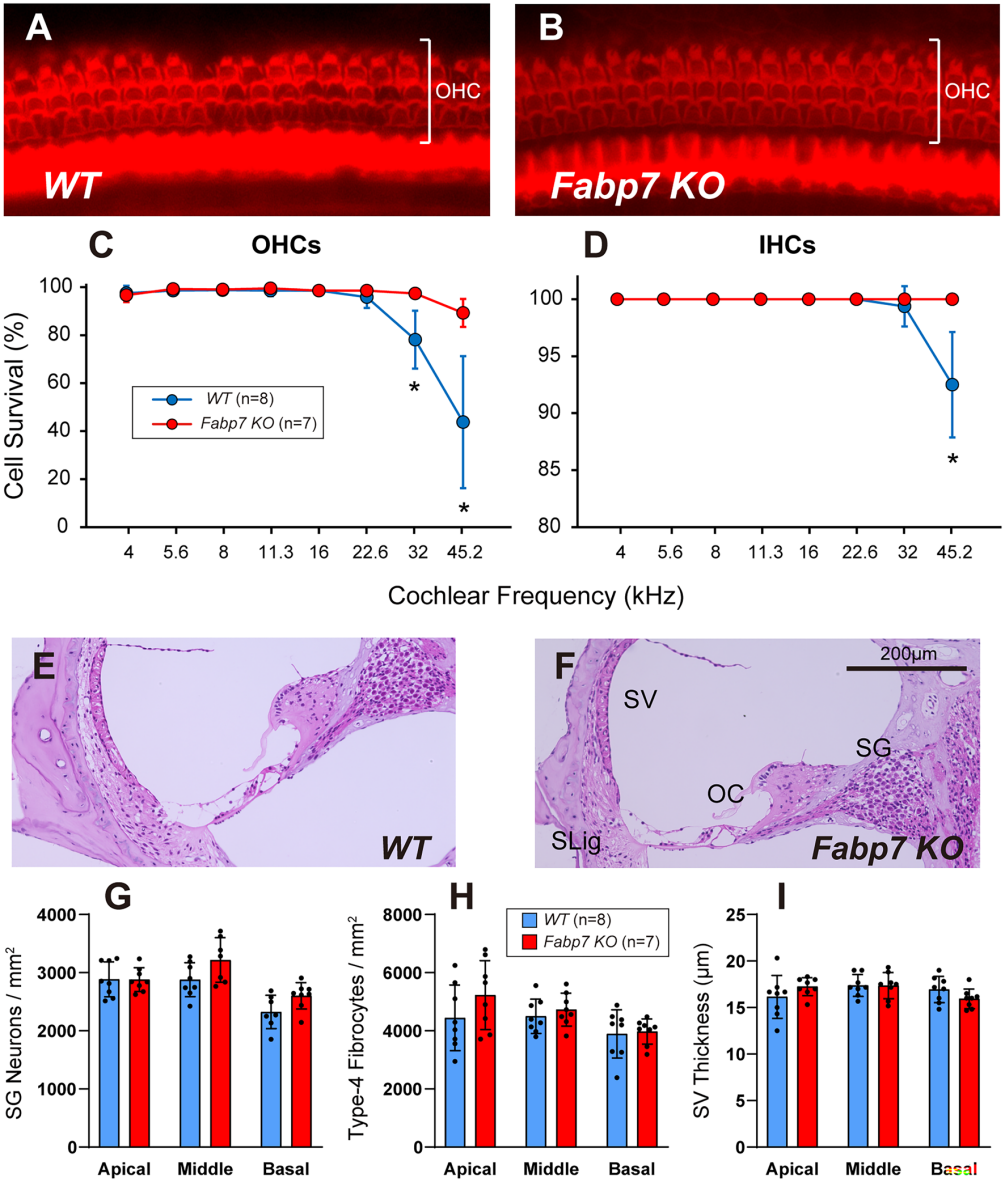
Histological analyses of the cochlea 14 days after noise exposure. Representative images of the organ of Corti (OC) in the 32-kHz region of WT mice (**A**) and *Fabp7* KO mice (**B**) on post-noise exposure day 14. Outer hair cell (OHC) and inner hair cell (IHC) survival in *Fabp7* KO mice was significantly higher than that in WT mice in the 32- and 45.2-kHz regions and 45.2-kHz regions, respectively (**C, D**). Representative images of the cochlear middle turn of WT (**E**) and *Fabp7* KO mice (**F**) on post-noise exposure day 14. There were no apparent differences in cochlear appearance between the two groups. Quantitative data for spiral ganglion (SG) neuron count (**G**), type-4 fibrocyte count (**H**), and stria vascularis (SV) thickness were not significantly different between the two groups. Statistical significance was determined using two-way analysis of variance, followed by Šídák multiple comparison test. Error bars represent standard deviation. Spiral ligament; SLig.

Noise exposure causes various types of cochlear damages including loss of important cells such as hair cells and SG neurons, and atrophy of tissues such as a stria vascularis (SV). To clarify the morphological basis of the protective effect of *Fabp7* deletion, histological examinations were performed using cochlear wholemounts and paraffin sections for WT mice (n = 8) and *Fabp7* KO mice (n = 7) after final ABR measurements (Fig. 1A). The inner hair cells (IHCs) and outer hair cells (OHCs) were counted using whole mounts stained with rhodamine-conjugated phalloidin (Fig. 3A,B). Quantitative analyses revealed that OHC survival at 32 and 45.2 kHz regions and IHC survival at 45.2 kHz region were significantly higher in *Fabp7* KO mice than those in the WT mice (Fig. 3C,D). The number of SG neurons and type-4 fibrocytes, as well as SV thickness were evaluated using cochlear coronal paraffin sections (Fig. 3E,F). No significant differences in SG neuron survival, type-4 fibrocyte survival, or SV thickness were observed after noise exposure between WT and *Fabp7* KO mice (Fig. 3G–I). These results suggested that *Fabp7* deletion protected against hair cell loss after noise exposure at high frequencies, supporting the results of ABR measurements.

### Lipid peroxidation product is affected by Fabp7 deletion

**Figure 4:**
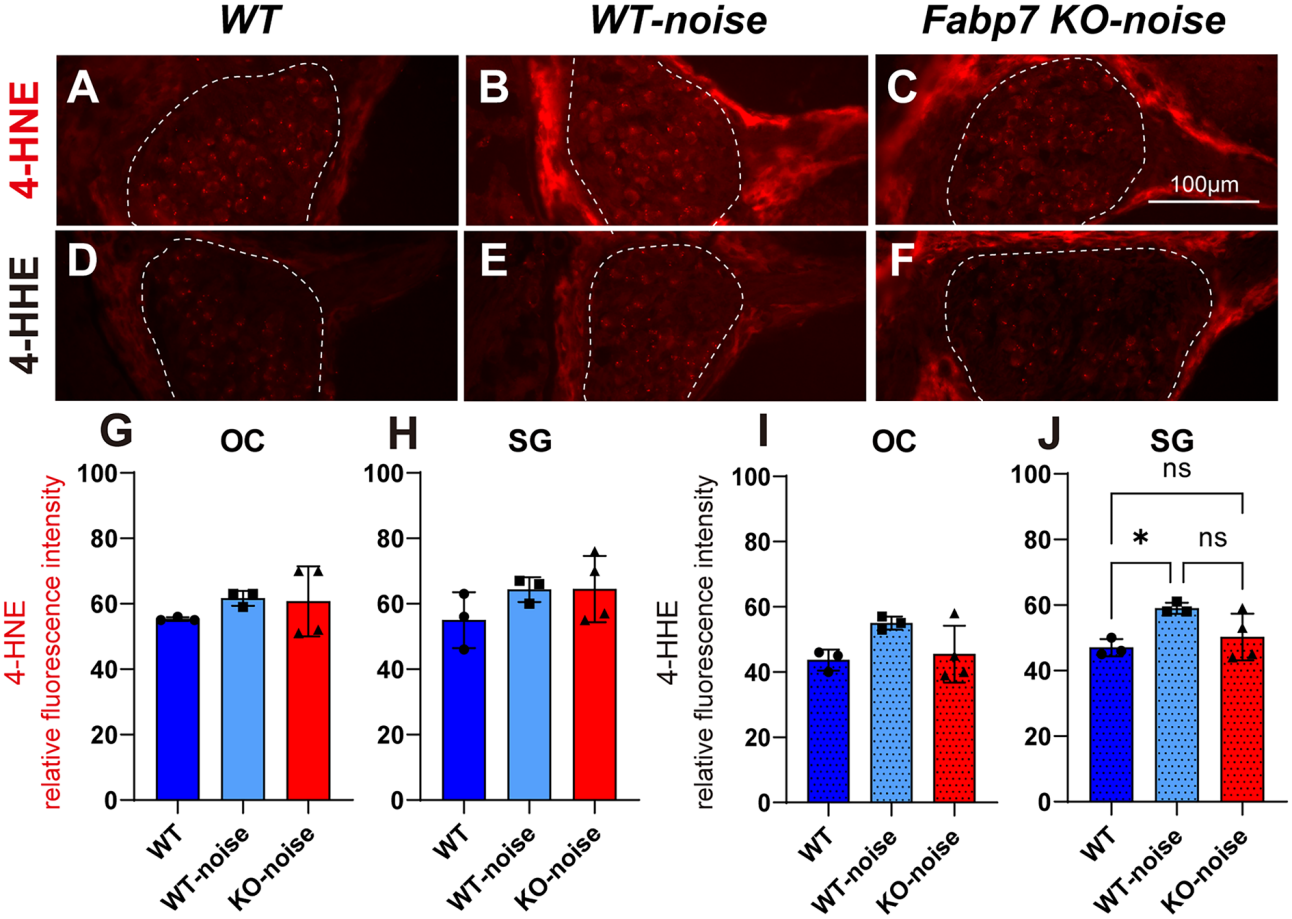
Immunohistochemistry of oxidized fatty acids in the cochlea at post-noise exposure day 1. 4-Hydroxy-2-nonenal (4-HNE) (**A–C**) and 4-hydroxy hexenal (4-HHE) (**D–F**) immunostaining in the SG of WT mice (**A, D**), WT mouse one day after noise exposure (**B, E**), and *Fabp7* KO mice one day after noise exposure (**C, F**). Quantification of 4-HNE relative fluorescence intensity in the OC (**G**) and SG (**H**). Quantification of 4-HHE relative fluorescence intensity in the OC (**I**) and SG (**J**). 4-HHE immunofluorescence was significantly increased in noise-exposed WT mice compared to normal WT mice, but not in noise-exposed *Fabp7* KO mice (**J**). Statistical significance was determined using a one-way analysis of variance followed by Tukey’s multiple comparison test. n.s., not significant; *P < 0.05. Error bars represent standard deviation.

To evaluate oxidative damage in the cochlea after noise exposure, we semi-quantified the immunoreactivities of 4-Hydroxy-2-nonenal (4-HNE), a major product of lipid peroxidation^29^ and 4-hydroxy hexenal (4-HHE), a lipid peroxidation product derived from oxidized n-3 fatty acids, such as DHA^30^ (Fig. 1A). We assessed 4-HNE and 4-HHE expression in the OC and SG of WT mice (n = 3) without noise exposure and in WT (n = 3) and *Fabp7* KO mice (n = 4) one day after noise exposure at 10 weeks of age (Fig. 4A–F). Quantitative analysis indicated that 4-HNE immunofluorescence in the OC and SG (Fig. 4G,H) and 4-HHE immunofluorescence in the OC (Fig. 4I) did not differ significantly among the three groups. In contrast, 4-HHE immunofluorescence in the SG was significantly higher in noise-exposed WT mice than in control WT mice, but not in noise-exposed *Fabp7* KO mice (Fig. 4J). Thus, expression of the oxidized n-3 fatty acid-mediated lipid peroxidation product was affected by *Fabp7* deletion.

### Transcriptome analysis of differentially expressed genes in the WT and Fabp7 KO mouse inner ear

**Figure 5:**
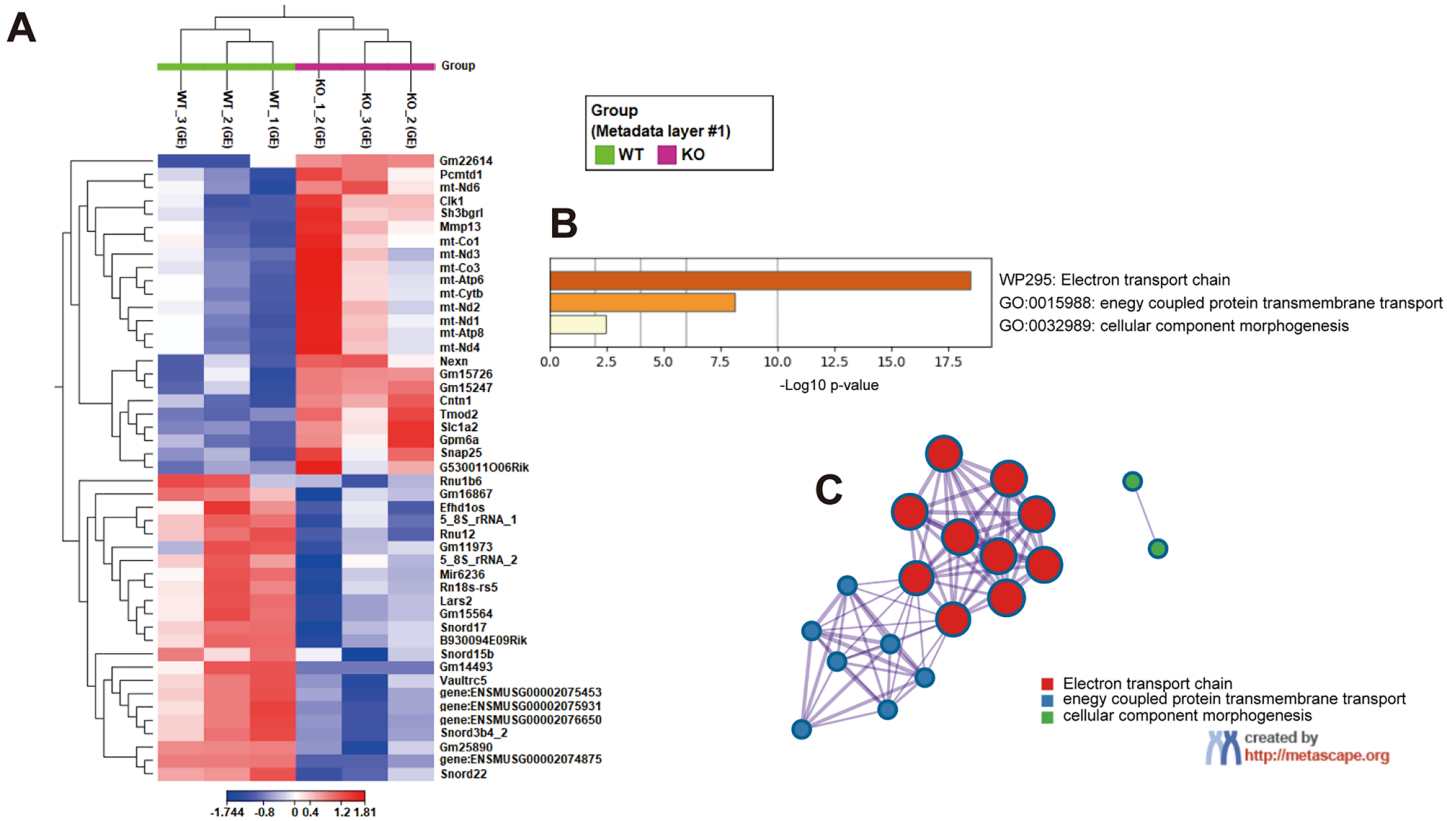
Enrichment analysis of differentially expressed genes in the inner ear at post-noise exposure day 1, using Metascape. Heatmap of 24 upregulated and 23 downregulated differentially expressed genes (DEGs) in *Fabp7* KO mice (Max RPKM group mean > 1, false discovery rate < 0.05) (**A**). The bar graph shows enriched ontology clusters of 24 upregulated DEGs in *Fabp7* KO mice, revealing the increased expression of electron transport chain-mediated genes (**B**). Network layout of significantly enriched terms obtained using Metascape (**C**).

To elucidate the function of FABP7 in the inner ear, particularly in the context of its protective mechanisms against noise-induced trauma, we evaluated gene expressions between WT (n = 3) and *Fabp7* KO mice (n = 3) one day after noise exposure, using RNA sequencing analysis (Fig. 1A). The read number was approximately 27–32 million paired-end reads per sample. A total of 24 and 23 genes were identified as upregulated and downregulated differentially expressed genes (DEGs), respectively, in *Fabp7* KO mice (Supplementary Tables 1 and 2). Notably, the upregulation of genes related to nicotinamide-adenine dinucleotide (NADH) dehydrogenase (*mt-Nd1, 2, 3, 4, and 6*) and a glial high-affinity glutamate transporter (*solute carrier family 1, member 2: Slc1a2*) and downregulation of genes related to C/D box small nucleolar RNAs were observed in *Fabp7* KO mice. A heat map of these genes is shown in Fig. 5A.

### Bioinformatics analysis of DEGs in the WT and Fabp7 KO mouse inner ear

Functional annotation and pathway enrichment analyses of the DEGs were performed using Metascape^31^. We found no significantly enriched terms in the downregulated DEGs in *Fabp7* KO mice. In contrast, “electron transport chain” was the significantly enriched term in the upregulated DEGs in *Fabp7* KO mice (Fig. 5B,C). Thus, the expression of mitochondrial function-mediated genes was upregulated or less impaired after noise exposure in the inner ear of *Fabp7* KO mice, compared to that in WT mice, implying that mitochondrial activities were more preserved after noise exposure in the cochlea of *Fabp7* KO mice.

### Identification of variable metabolites in the WT and Fabp7 KO mouse inner ear

To identify the changes in metabolites induced by *Fabp7* deletion in the inner ear after noise exposure, we performed metabolomic analysis^32–34^ using WT and *Fabp7* KO mouse inner ear one day after noise exposure (a total of 15 analyses using 5 samples for each group) (Fig. 1A). We found 162 metabolites with increased levels and 294 metabolites with reduced levels reliably annotated in *Fabp7* KO mice (Supplementary Tables 1 and 2); the data was populated in the enrichment analysis of MetaboAnalyst^35^.

### Bioinformatics analysis of variable metabolites in the WT and Fabp7 KO mouse inner ear

**Figure 6:**
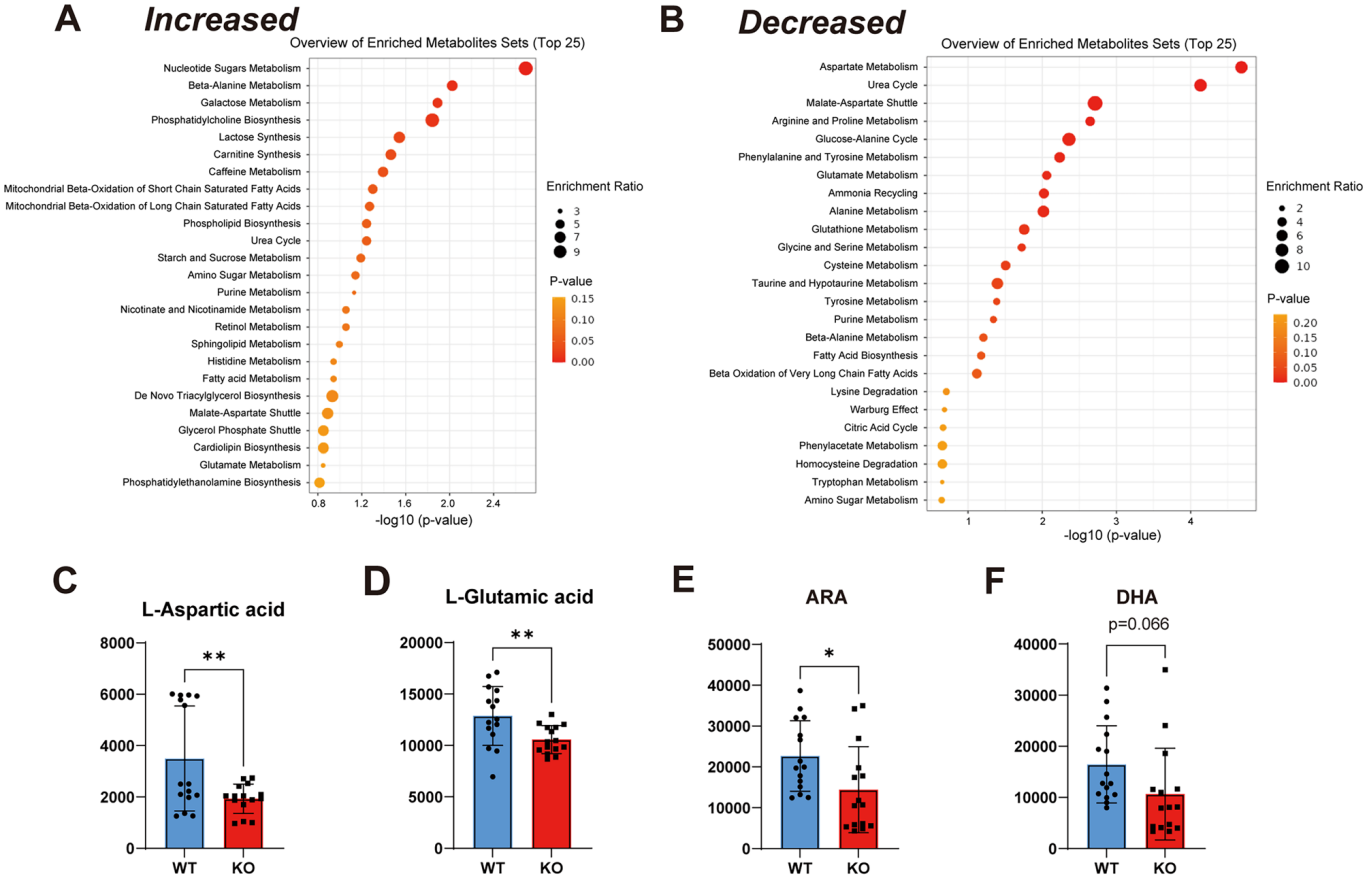
Metabolic analysis using the inner ear one day after noise exposure. Overview of increase-enriched metabolite sets (**A**) and decrease-enriched metabolite sets (**B**) in *Fabp7* KO mice compared to WT mice analyzed using MetaboAnalyst. Quantitative data of representative metabolites: l-aspartic acid (aspartate) (**C**), l-glutamic acid (glutamate) (**D**), arachidonic acid (ARA) (**E**), and docosahexaenoic acid (DHA) (**F**). Statistical significance was determined using an unpaired t-test. *P < 0.05; **P < 0.01. Error bars represent standard deviation.

After converting the names of the metabolites to registered names in the latest version of MetaboAnalyst, we finally selected 47 metabolites with increased levels and 67 metabolites with reduced levels in *Fabp7* KO mice (Supplementary Tables 3 and 4). We performed functional annotation and pathway enrichment analysis using MetaboAnalyst with the small molecule pathway database^36^. As a result, metabolites associated with “nucleotide sugars metabolism”, “beta-alanine metabolism”, “galactose metabolism,”, and “phosphatidylcholine biosynthesis” were the significantly enriched terms for increased metabolites in *Fabp7* KO mice (Fig. 6A). In contrast, metabolites associated with “aspartate (l-aspartic acid) metabolism”, “urea cycle”, “malate-aspartate shuttle”, and “glutamate (l-glutamic acid) metabolism” were the significantly enriched terms for reduced metabolites in *Fabp7* KO mice (Fig. 6B). Quantitative data for representative reduced metabolites in *Fabp7* KO mice are shown in Fig. 6C–F. Aspartate and glutamate, representative excitatory transmitters, were significantly lower in the *Fabp7* KO mice (Figs. 6C,D, respectively), and ARA, an essential precursor of eicosanoids, were also significantly reduced in *Fabp7* KO mice. Collectively, noise exposure induced various metabolic changes such as amino acid and lipid metabolism in the inner ear of *Fabp7* KO mice compared to those in WT mice; these metabolic changes may contribute to the hearing-preservation phenotype of *Fabp7* KO mice.

## Discussion

In this study, we examined the impact of *Fabp7* deficiency on hearing following exposure to noise using newly developed genome-edited *Fabp7* KO mice with a C57BL/6 background. Our findings demonstrated that *Fabp7* deletion remarkably reduced the permanent threshold elevation after noise exposure, thereby preserving the integrity of OHCs and IHCs, particularly in high-frequency regions. Moreover, *Fabp7* KO mice exhibited no increase in the levels of toxic oxidized lipids and displayed upregulated or less impaired gene expression associated with electron transport chain function after noise exposure, further substantiating the protective effects associated with *Fabp7* deletion. Furthermore, metabolomic analysis revealed notable alterations in the levels of some metabolites, including excitatory neurotransmitters such as glutamate and aspartate.

FABP7 is essential for neuronal and glial cell development, as well as postnatal neural stem cell proliferation, highlighting its importance in the nervous system^23,37–40^ and the adverse effects of its deficiency. For example, *Fabp7* KO mice have exhibited lower survival of ventral neurons and poorer motor function recovery than WT mice in a spinal cord injury model^41^. *Fabp7* KO mice also exhibit reduced neurogenesis in the dentate gyrus under physiological and ischemia-induced conditions^39^. In contrast, our study revealed prospective effects of *Fabp7* deficiency on cochlear function following noise trauma. Similarly, *Fabp7* KO mice have exhibited better final clinical scores than those of WT mice in an experimental autoimmune encephalomyelitis model, although an early deterioration in clinical scores has also been noted^42^. Other studies have also suggested positive effects of FABP7 deficiency on neurological prognosis; FABP7 upregulation induces a neurotoxic phenotype in an astrocyte-motor neuron co-culture model^43^, and the administration of an FABP inhibitor reduces brain infarct volumes and neurological deficits in a mouse cerebral ischemia–reperfusion injury model^44^. Our findings further support the notion that FABP7 exacerbates nerve tissue damage, suggesting a dual role for FABP7 during neurogenesis and physiological states versus tissue damage. This duality might be associated with the involvement of FABP7 in the external stimulus response of astrocytes through the regulation of Caveolin-1 expression, which affects lipid raft function^45^. Inflammation is a consequential response to external stimuli, and FABP7 overexpression promotes proinflammatory responses in mouse astrocytes^43^. However, *Fabp7* KO mice also exhibit elevated mRNA expression levels of inflammatory cytokines in the spinal cord after inducing experimental autoimmune encephalomyelitis^42^. Thus, FABP7 deficiency elicits contrasting reactions under various conditions. Although our RNA sequencing and metabolomic analyses did not directly indicate the suppression of inflammatory reactions, noise-exposed *Fabp7* KO mice showed a significant decrease in the levels of ARA, a precursor of inflammatory bioactive lipids and eicosanoids. Further investigations into the relationship between noise vulnerability and PUFA metabolites may clarify the pathology of NIHL.

Detrimental effects of noise trauma on the cochlea include mechanical damage to the cochlear structure, reduced blood flow, sterile inflammation, oxidative stress-induced mitochondrial dysfunction, apoptosis, and glutamate excitotoxicity. These pathologies collectively contribute to the development of PTS or temporary threshold shift, depending on severity of the injury^3,7,46^. Our current study primarily focused on improving hearing ability and cochlear pathology 14 days following acoustic exposure, and assessing its impact on PTS. Interestingly, there were no significant differences in ABR thresholds one day after noise exposure, indicating comparable cochlear damage in both WT and *Fabp7* KO mice. However, based on the distinct differences between the two groups observed 14 days following exposure, we hypothesized that *Fabp7* deletion mitigates the delayed-onset of harmful effects, including oxidative stress, inflammation, and apoptosis. The expansion of OHC apoptosis and the immune response of cochlea, such as increase in macrophages and neutrophils, occurs several days following noise exposure^47–49^, suggesting that *Fabp7* deletion may alleviate these harmful reactions. Although we did not confirm changes in the expression of pro-inflammatory and apoptosis-related genes, reduction in the levels of oxidized lipids and PUFAs in *Fabp7* KO mice supports our hypothesis as these compounds can induce oxidative stress and inflammation^11^. Here, we observed the mitigation of ABR threshold shifts at 8, 12, and 32 kHz in *Fabp7* KO mice after noise exposure, but the underlying increase in OHC survival was only confirmed in the 32 kHz region in cochlear wholemounts. Since a loss of stereocilia tip links and metabolic disturbance in the cochlear lymph fluid can cause PTS^3^, these injury mechanisms may contribute to the change in ABR threshold shifts at 8 and 12 kHz in *Fabp7* KO mice.

Interestingly, genes related to the electron transport chain were upregulated or less impaired in the inner ear of noise-exposed *Fabp7* KO mice, implying preserved mitochondrial function. Mitochondria play a role in metabolic damage and ROS generation induced by noise trauma^50,51^; severe mitochondrial damage can trigger mitochondria-mediated apoptosis^52^. Mice lacking NADH dehydrogenase (ubiquinone) Fe–S protein 4, a subunit of the mitochondrial complex I, have displayed accelerated permanent low-frequency threshold shifts following moderate noise exposure^53^. Although there is no direct evidence of the mitochondrial protective effect of *Fabp7* deficiency, we observed increased expression of genes related to NADH dehydrogenase in noise-exposed *Fabp7* KO mouse cochlea. Further investigations focusing on mitochondrial function and detailed mitochondrial histology will contribute to elucidating the mechanism underlying this effect in future studies.

Glutamate excitotoxicity, a cell death mechanism triggered by excessive glutamate release from neurons and glial cells, has been implicated in various neurological disorders^54^. Excess glutamate can lead to glutathione deficiency, sustained activation of the glutamate receptor in the postsynaptic membrane, and damage to afferent neural fibers^8,55^. The accumulation of glutamate is associated with acute and chronic deterioration of the auditory nervous system, wherein acoustic overstimulation induces glutamate-induced excitotoxicity in IHCs and type 1 SG neurons^8,56–59^. Our study observed an improved survival rate of hair cells in *Fabp7* KO mice, possibly due to a significant decrease in glutamate levels within the cochlea in *Fabp7* KO mice, compared to those in WT mice. Although FABP7 is expressed in many glial cells of the nervous system and participates in modulating the signaling pathways of glutamatergic synapses, the direct relationship between FABP7 and glutamate metabolism remains unclear. In this study, the expression of *Slc1a2*, the principal transporter participating in the clearance of glutamate, was increased in the cochlea of noise-exposed *Fabp7* KO mice. Interestingly, a recent study has demonstrated a significant decrease in *Slc1a2* gene expression in the cochlea 15 days after noise exposure compared to those in control conditions and the first day after noise exposure. These findings emphasize the importance of *Slc1a2* in the pathology of noise-induced trauma^60^. Thus, *Fabp7* deficiency leads to an otoprotective phenotype, possibly through the increased expression of glutamate transporters and reduced glutamate levels. We anticipate that further research will advance our understanding of the underlying mechanisms, based on the results of this study.

In this study, we conducted metabolomic analysis of the noise-exposed inner ear tissue. Metabolomics is a state-of-the-art technique enabling the comprehensive analyses of all metabolites in a sample, providing a snapshot of its physiological state^61^. However, research on the metabolome of inner ear pathologies remains limited^61,62^, with only a few published studies specifically focusing on mouse samples following noise exposure^63,64^. One such study by Ji et al. investigated the effect of noise exposure on metabolites in the entire inner ear of CBA/CaJ mice and revealed changes such as increased glutamate and aspartate levels^63^. Similar to glutamate, aspartate is an excitatory transmitter in the brain, which is released into the cochlea following noise exposure^65,66^. Interestingly, we observed a significant decrease in glutamate and aspartate levels in the inner ear of noise-exposed *Fabp7* KO mice, suggesting a potential role for FABP7 in mitigating excitotoxicity and preserving hearing. Thus, glutamate and aspartate may be critical molecules involved in noise-induced cochlear damage, with FABP7 potentially modulating their levels directly or indirectly. Additionally, we identified other changes, including an increase in nucleotide sugar metabolism, in the inner ear metabolome of noise-exposed *Fabp7* KO mice. However, it remains unclear whether these metabolite alterations indicate tissue damage or reparative processes. Further advancements in inner ear metabolomic analyses will provide a more comprehensive understanding of these intricacies.

This study has several limitations. First, we utilized whole inner ear tissue for RNA sequencing and metabolomic analyses. While these methods allow the assessment and quantification of considerable changes in gene expression and metabolites within inner ear tissues, they did not provide detailed insights into cellular changes. In addition, the quality of RNA in this study was sub-optimal. Fortunately, single-cell RNA sequencing of the mouse inner ear has been performed previously, and a cell-type-specific transcriptomic map of the cochlear response to acoustic overexposure is available^67^. Further investigations should leverage this novel technique for a more comprehensive evaluation. Similarly, metabolomic analysis is still a developing field, and currently, analyses using the entire mouse inner ear are appropriate because of the widespread impact of noise exposure on metabolomes across the intracellular and extracellular inner ear compartments. Second, we used genome-edited *Fabp7* KO mice with a C57BL6 background. Hence, the developmental influence of *Fabp7* deletion may affect the results of noise exposure experiments initiated at nine weeks of age. Although no difference in ABR thresholds was observed between WT and *Fabp7* KO mice prior to noise exposure, elucidating the role of FABP7 in the defense mechanism against noise trauma in adult mice would necessitate experiments using *Fabp7* conditional KO mice or the local application of FABP7 inhibitors. Notably, a recent report demonstrated the ameliorative effects of an FABP7 inhibitor on oligodendrocyte injury in a mouse model of multiple sclerosis^68^ and on cerebellum dysfunction in a multiple-system atrophy mouse model^69^. Therefore, it would be worthwhile to explore whether such an FABP7 inhibitor could play a protective role against cochlear noise trauma. Third, the protective effect of *Fabp7* deletion on hair cells is indirect because it is not expressed in hair cells. Moreover, detailed changes in support cells lacking FABP7 were not extensively evaluated in this study despite the lack of noticeable differences in supporting and non-neuronal cells observed in cochlear sections stained with hematoxylin and eosin (H&E). Future investigations could benefit from employing sophisticated techniques such as primary culture and single-cell RNA sequencing to enable more detailed analyses. Finally, although the gene expression associated with electron transport chain function after noise exposure was upregulated or less impaired in *Fabp7* KO mice, this change does not necessarily indicate preservation of mitochondrial function. Evaluation of mitochondrial functions, such as the oxygen consumption rate, using cochlear tissues is yet to be established and remains a future challenge.

In conclusion, our study highlights the benefits of FABP7 deficiency in mitigating cochlear damage following noise exposure. FABP7 expression is specific to non-hair and non-neuronal cells in the cochlea. The observed protective effect of its deletion was supported by the upregulated or less impaired expression of genes involved in the electron transport chain, as well as alterations in several metabolites, including excitotoxic glutamates and aspartates. Notably, FABP7 plays a crucial role in signal regulation in response to external stimuli and may serve as a vital molecule in the mechanisms underlying nervous system trauma, including noise trauma. These findings contribute to our understanding of the complex processes involved in cochlear injury and highlight the potential therapeutic significance of targeting FABP7 in noise-induced damage. Further research in this field may open new avenues for the development of novel strategies to prevent or treat NIHL.

## Methods

### Animals and experimental protocol

WT and *Fabp7* KO mice with a C57BL/6NCrl genetic background, generated using the CRISPR-Cas9 nickase system, were used^28^. Heterozygous *Fabp7* KO mice were crossed, and their offspring were genotyped using polymerase chain reaction (PCR) with the following primers: *Fabp7* forward (5′- GGTAAGACCCGAGTTCCTCCAGTT -3′) and *Fabp7* reverse (5′-TGAGTGGGCTATGCACAGTCTT-3′). The following cycling conditions were used: 94 °C for 3 min, 35 cycles of 94 °C for 30 s, 58 °C for 30 s, and 72 °C for 30 s, followed by 72 °C for 3 min and 4 °C indefinitely. The PCR products were separated on a 2.2% agarose gel to detect bands of the following expected sizes: 271 bp for the WT and 243 bp for *Fabp7* mutant alleles. After obtaining the first generation of WT and homozygous *Fabp7* KO mice, crosses were made between each group and their male children were used in the experiment. The mice were housed under a 12 h light–dark cycle at a constant temperature with ad libitum access to food and water. All mice were treated following the guidelines presented in The Standards for Human Care and Use of Laboratory Animals of Tohoku University, Guidelines for Proper Conduct of Animal Experiments by the Ministry of Education, Culture, Sports, Science, and Technology of Japan, and the National Institute of Health Guide for the Care and Use of Laboratory Animals. Animal Research: Reporting of In Vivo Experiments (ARRIVE) guidelines were followed. All animal experiments were approved by the Ethics Committee for Animal Experiments of the Tohoku University Graduate School of Medicine (2020-132).

Figure 1A shows a schematic representation of the experimental design. Initial ABR measurements were performed at 9 weeks of age and noise exposure was applied at 10 weeks of age in 8 male WT and *Fabp7* KO mice. A second ABR measurement was performed 24 h after noise exposure, with the final measurement and sampling conducted at 12 weeks. Because the cochleae of a *Fabp7* KO mouse were damaged during sampling, histological analysis for *Fabp7* KO mice was performed on 7 animals. For the analysis of RNA sequencing, 3 WT and *Fabp7* KO mice were sampled 24 h after noise exposure at 10 weeks. For the analysis of metabolomics, 5 WT and *Fabp7* KO mice were sampled 24 h after noise exposure at 10 weeks. For the histological analysis of oxidative stress, 3 control WT mice, 3 noise-exposed WT mice, and 4 noise-exposed *Fabp7* KO mice were sampled 24 h after noise exposure at 10 weeks.

### *Cdh23* genotyping

Genomic DNA was isolated from WT and *Fabp7* KO mice (n = 5 per group) and used as a template to amplify 360-bp regions around the 753rd nucleotide in the *Cdh23* gene using PCR. The resultant products were then sequenced^70^ using the following primers: *Cdh23* forward 5′-GATCAAGACAAGACCAGACCTCTGTC-3′ and *Cdh23* reverse 5′-GAGCTACCAGGAACAGCTTGGGCCTG-3′. The cycling conditions were as follows: 95 °C for 2 min, 35 cycles of 95 °C for 30 s, 60 °C for 1 min, 72 °C for 1 min, and 72 °C for 5 min.

### Cochlear function testing

The ABRs were recorded as described previously^26^. Mice were anesthetized using an intraperitoneal injection of ketamine (100 mg/kg body weight) and xylazine (20 mg/kg body weight). Needle electrodes were placed subcutaneously at the vertex (active), the base of the pinna (reference), and the back (ground). ABR recordings were performed using the TDT System 3 auditory-evoked potential workstation and BioSigRP software (Tucker-Davis Technologies, Alachua, FL). ABRs were evoked using bursts of pure tone at frequencies of 4, 8, 12, 16, and 32 kHz. The evoked responses were averaged using 1,000 responses for each SPL. Responses were collected for stimulus levels in 5-dB steps from 100 dB SPL to 10 dB SPL. The ABR threshold was defined as the lowest sound level at which a reproducible waveform was confirmed; it was determined by a separate examiner blinded to the genotype. If a mouse showed no response, the stimulus threshold was defined as 105 dB SPL.

### Noise exposure

Awake and unrestrained mice were placed in a wire-mesh cage and exposed to 8–16 kHz octave-band noise at 100 dB SPL for 2 h in a sound-exposure box as previously described^53^. Sound was generated using a waveform generator (SF-06, Random Noise Generator; RION), amplified (D-75A; Crown), filtered (Multifunction Filter; NF Corporation), and delivered by a loudspeaker (2446H; JBL) positioned at the center of the cage. The sound levels were measured using a sound level meter (2250 L; Brüel & Kjær.)

### Tissue preparation

Mice were deeply anesthetized with an intraperitoneal injection of ketamine (100 mg/kg) and xylazine (20 mg/kg), and transcardially perfused with 10% formalin neutral buffer solution (pH 7.4) (Wako, Osaka, Japan). The inner ear was quickly removed and immersed in 10% formalin-neutral buffer. Small holes were made in the round and oval windows and the apex of the cochlea. The cochleae were perfused through the cochlear scalae, post-fixed with 10% formalin neutral buffer solution at 4 °C for 16 h, and decalcified in 10% ethylenediaminetetraacetic acid for 2 days at room temperature.

### Cochlear wholemount and hair cell counts

After fixation and demineralization, cochlear wholemount was performed as previously described^13^. Hair cells were stained for F-actin using rhodamine-conjugated phalloidin (1:100, Rhodamine Phalloidin, Thermo Fisher Scientific, Waltham, MA, USA) for 1 h at room temperature. Low-power fluorescence images were obtained using a microscope (BZ-9000, Keyence, Osaka, Japan) and BZ-H1 software (Keyence), and full-length cochlear images were assembled and analyzed using Adobe Photoshop. The ImageJ plug-in was used to create a cochlear frequency map. We counted the losses of IHCs and OHCs in seven frequency-specific regions (4.0, 8.0, 11.3, 16.0, 22.6, 32.0, and 45.2 kHz) of the cochlea. We observed 60 OHCs associated with 20 IHCs using a fluorescence microscope (BZ-9000) with a 40× objective.

### Cochlear histology using coronal sections

To detect differences in cochlear histological damage after noise exposure, paraffin Sections (3 μm) were stained with H&E and observed under a light microscope (BZ-9000) separately in the apical, middle, and basal turns. The cell density of SG neurons, type-4 fibrocytes, and the thickness of the SV within each turn were calculated using a 40× objective. The area and cell numbers were determined using the BZ-H1C software. Cell density was calculated by dividing the total number of cells by the measured area and expressed as the number of cells per mm^2^. For SV thickness, three measurements from the endolymphatic surface of the marginal cells to the spiral ligament side of the basal cells were obtained from each image and averaged. Two sections per animal were used to calculate mean values.

### Immunohistochemistry of frozen sections

To prepare frozen sections, decalcified cochleae were treated with 30% sucrose in phosphate-buffered saline (PBS), embedded in Tissue-Tek optimum cutting temperature compound (Sakura Finetek, Tokyo, Japan), frozen, and cut at 10 µm thickness parallel to the modiolus using a CM3050 cryostat (Leica Biosystems, Wetzlar, Germany). Sections were mounted on MAS-coated glass slides (Superfrost; Matsunami Glass, Kishiwada, Japan). After rinsing with PBS, tissue sections were blocked with 3% bovine serum albumin/0.3% Triton X-100/ PBS for 30 min at room temperature, the unconjugated AffiniPure Fab fragment of anti-mouse IgG (1:10, Jackson ImmunoResearch, West Grove, PA) for 2 h at room temperature, and incubated overnight with the following primary antibodies: (1) rabbit IgG anti-FABP7 (1:1000, Merck, Darmstadt, Germany #PRS4259), (2) mouse IgG anti-TUJ-1 (1:500, Abcam, Cambridge, UK #ab78078), (3)　mouse IgG2a anti-Myosin 7a (MYO7a) (1:200, Santa Cruz Biotechnology, Dallas, TX, USA #SC-74516), and (4) goat IgG anti-SOX2 (1:500, R&D Systems, Minneapolis, MN, USA #AF2018). Following overnight incubation with the primary antibodies at 4 °C, the sections were rinsed and then incubated with donkey anti-rabbit IgG Alexa Fluor 568 conjugate (1:500, Thermo Fisher Scientific, Waltham, MA, USA #A-10042) and goat anti-mouse IgG2a Alexa Fluor 488 conjugate (1:500, Thermo Fisher Scientific #A-21131), or chicken anti-rabbit IgG Alexa Fluor 647 conjugate (1:500, Thermo Fisher Scientific #A-21443) and donkey anti-mouse IgG Alexa Fluor 568 conjugate (1:500, Thermo Fisher Scientific #A-10037) and donkey anti-goat IgG Alexa Fluor 488 conjugate (1:500, Thermo Fisher Scientific #A-11055) for 1 h at room temperature. The slides were mounted with coverslips using a mounting medium with 4′,6-diamidino-2-phenylindole (VECTASHIELD Mounting Medium with DAPI, Vector Labs #H-1200). For the evaluation of FABP7 expression in the cochleae of WT and *Fabp7* KO mice, the slides were imaged using a fluorescence microscope (BZ-9000) and BZ-H1C software for the same exposure time at 20× magnification. For the detailed localization analysis of FABP7 in the cochlea of WT mice, a confocal microscope (TCS SP8, Leica Microsystems, Wetzlar, Germany) fitted with a 63× objective was used. Maximum intensity projection images were created using an image processing software (Leica Application Suite X, Leica Microsystems). The images were merged using Adobe Photoshop (San Jose, CA, USA).

### Immunostaining of oxidized fatty acid in cochlear sections

Frozen sections were prepared as described above. After rinsing with PBS, antigen retrieval was performed using microwave irradiation for 15 min in 0.05% citraconic anhydride buffer (Wako #097-06192). Tissue sections were blocked with 3% bovine serum albumin/0.3% Triton X-100/PBS for 30 min at room temperature and incubated overnight with the following primary antibodies: (1) mouse IgG1 anti-4-NHE (1:200, JaICA, Fukuroi, Japan #MHN-100P), (2)　mouse IgG1 anti-4-HHE (1:100, JaICA #MHH-030n). Following overnight incubation with the primary antibodies at 4 °C, the sections were rinsed and then incubated with goat anti-mouse IgG1 Alexa Fluor 568 conjugate (1:1000, Thermo Fisher Scientific #A-21124) for 1 h at room temperature. The nuclei were then counterstained with 4′,6-diamidino-2-phenylindole (1:1000; Merck, Darmstadt, Germany #9542), and the slides were mounted with coverslips using a mounting medium (VECTASHIELD Mounting Medium, Vector Labs #H-1000). The slides were imaged using a fluorescence microscope (BZ-9000) for the same exposure time at 40× magnification. The areas and signal intensities of the OC and SG regions were measured at the middle turns using the BZ-H1C analysis software. Two sections per mouse were analyzed and used to calculate the mean values.

### RNA extraction and RNA sequencing

RNA extraction and sequencing were performed as previously described^53^. The inner ears were quickly dissected from the skull and stored at -80 ℃. RNA extraction was performed using RNAiso Plus (Cat# 9180; Takara Bio, Kusatsu, Shiga, Japan), according to the manufacturer’s instructions, with freeze-crushing of the tissues. RNA pellets were dissolved in 30 µL Milli-Q Water (Merck Millipore, Burlington, MA, USA). The RNA integrities of eight samples (WT: *n* = 3 and *Fabp7* KO: *n* = 3) were determined using the Agilent RNA 600 Nano Kit (Cat# 5067-1511; Agilent Technologies, Santa Clara, CA, USA) on a Bioanalyzer (Agilent Technologies). The mean RNA integrity of all samples was 4.85 ± 0.85. Subsequently, 500 ng of RNA from each sample was used to create libraries using the NEBNext Ultra II RNA Library Prep Kit for Illumina and the NEBNext rRNA Depletion Kit v2 (Cat# E7770S and E7400L; New England Biolabs, Ipswich, MA, USA), according to the manufacturer’s instructions. The duration of the final PCR cycle was 15 min. The concentration and size distribution of the libraries were measured using the Agilent DNA 7500 kit (Cat#5067-1506; Agilent Technologies, Santa Clara, CA, USA) and a bioanalyzer (Agilent Technologies). All samples were analyzed using next-generation sequencing (NGS) equipment. The libraries were pooled and the concentrations were adjusted to 1 nM. The pooled libraries were subjected to denaturation and neutralization assays. Subsequently, the libraries were diluted to 1.8 pM and subjected to NGS run using NextSeq500/550 v2.5 (75 cycles) kits (Cat#20024906; Illumina, San Diego, CA, USA) in the NextSeq 500 System (Illumina). Sequencing was performed using paired-end reads of 36 bases. Following this, FASTQ files were exported, and basic information of the NGS run data was checked using the CLC Genomics Workbench software (version 22.0; QIAGEN, Hilden, Germany). During quality assessment of the reads, a Phred Score > 20 was confirmed for 99.7% of all reads, indicating success of the run.

### Bioinformatics analysis for RNA sequencing

The average reads per kilobase of exon per million mapped reads of > 1 and false discovery rate < 0.05 were regarded as DEGs and uploaded to Metascape (https://metascape.org/gp/index.html#/main/step1), which is an open online analysis software that can perform pathway and process enrichment analysis based on numerous ontology sources^31^.

### Metabolomics mass spectrometry

The inner ears were quickly harvested from the skull and stored at − 80 °C. Every tissue (approximately 10–50 mg) was placed in 1.5 mL screw tubes and a twofold (w/v) mixture of water/methanol (1:1, v/v) was added. Two pieces of 2 mm stainless beads were added to the tube, the mixture was shaken at 3,000 rpm for 5 min at room temperature using a beads crusher (TAITEC Co., Saitama, Japan), and centrifugated for 10 min at 15,000×*g* and 4 ℃.

To prepare study quality control (SQC) sample for metabolomics, every seven microliters of the supernatants were mixed. The SQC mixture or each sample was mixed with an equal volume of formic acid/water (0.1:100, v/v) and analyzed using liquid chromatography-mass spectrometry (LC–MS), wherein 1 μL of the sample aliquot was injected.

For LC–MS metabolomics, a TripleTOF 5600 quadrupole-time of flight hybrid mass spectrometer (SCIEX, Framingham, MA, USA) and a Nexera ultra-high-performance liquid chromatograph system (Shimadzu Co. Ltd., Kyoto, Japan) were used. Measurements were performed in both positive and negative ion modes. Semi-quantitative analysis by measurement and MS Peak processing were performed using Analyst TF version 1.6.0 (SCIEX) and MS-DIAL software^71^. For LC conditions in both positive and negative ion modes, a mixture of water: 1 M NH_4_HCO_3_ in water: 25% aqueous ammonium (99:1:0.1, v/v/v) was used as mobile phase A and acetonitrile was used as mobile phase B. The LC gradient flow was as follows: B (%), 95–5%; 0–10 min. The flow rate was set at 0.4 mL/min. An Agilent HILIC-Z column (150 mm × 2.1 mm i.d., 2.7 µm) was used and the column temperature was set to 40 ºC. Sample solution (1 μL) was injected and the gradient conditions were examined.

### Bioinformatics analysis for metabolomics

Raw sample data were deconvoluted, aligned, and annotated using MS-DIAL 4.9 (http://prime.psc.riken.jp/compms/msdial/main.html). The metabolites were annotated using the Human Metabolome Database (https://hmdb.ca/spectra/ms/search) and MS-DIAL databases. We performed a two-tailed t-test to identify metabolites with significant differences between WT and *Fabp7* KO mice using Microsoft Excel 2016. In each group, three analyses were performed with five samples each for a total of 15. Differential metabolites were defined as those with p-values < 0.05, and 456 reliably annotated metabolites were retained for downstream analysis. Enrichment analysis was performed using MetaboAnalyst 5.0 (https://www.metaboanalyst.ca/), a comprehensive web-based platform dedicated to metabolomic data analysis^35^.

### Statistical analyses

Statistical analyses were conducted using Prism 9 (GraphPad Software, San Diego, CA, USA) for the unpaired t-test, one-way analysis of variance (ANOVA) followed by Tukey’s multiple comparison test, and two-way or two-way repeated measures ANOVA followed by Šídák’s multiple comparison test. All data are presented as means ± standard deviation. Statistical significance was set at *P* < 0.05.

### Supplementary Information

Supplementary Information.

## Data Availability

The data generated and analyzed in the current study are available from the corresponding author upon reasonable request. The RNA sequencing data have been deposited in Gene Expression Omnibus (GSE241529).
