# Supplementary material for: Fatty acid binding protein type 7 deficiency preserves auditory function in noise-exposed mice
Source: Sci Rep. 2023 Dec 6;13:21494. doi: 10.1038/s41598-023-48702-4 (PMC10700610; doi:10.1038/s41598-023-48702-4)
Supplement: Supplementary file 1 — Supplementary Information. [file 41598_2023_48702_MOESM1_ESM.pdf]

# Supplementary data

**Supplementary Table 1. Twenty-four upregulated genes in the cochleae of *Fabp7* knockout mice**

RPKM: reads per kilobase of exon per million mapped reads; WT: wild type; KO: knockout; FDR: false discovery rate; MT: mitochondrial chromosome.

**Supplementary Table 2. Twenty-three downregulated genes in the cochleae of *Fabp7* knockout mice**

RPKM: reads per kilobase of exon per million mapped reads; WT: wild type; KO: knockout; FDR: false discovery rate; MT: mitochondrial chromosome.

**Supplementary Table 3. Forty-seven increased metabolites in the cochleae of *Fabp7* knockout mice**

HMDB, Human Metabolome Database, WT, wild type KO, knockout; NA, not applicable.

**Supplementary Table 4. Sixty-seven decreased metabolites in the cochleae of *Fabp7* knockout mice**

HMDB, Human Metabolome Database, WT, wild type KO, knockout; NA, not applicable.

**Supplementary Fig. 1. Histological images**

Sequence analysis of a single nucleotide polymorphism in the *Cdh23* gene. All wild-type and *Fabp7* knockout mice examined had the *Cdh23*$^{753A}$ genotype.

sTable 1. 24 up-regulated genes in the cochleae of Fabp7 knockout mice.

| Gene name | Description | Chromosome | RPKM | | P-value | FDR |
|---|---|---|---|---|---|---|
| | | | WT | KO | | |
| *Gm15726* | predicted gene 15726 | X | 0.732942 | 10.2454 | 7.4326E-10 | 2.3418E-06 |
| *Gm22614* | predicted gene 22614 | 3 | 0.965098 | 18.37967 | 3.3614E-08 | 7.3801E-05 |
| *G530011O06Rik* | RIKEN cDNA G530011O06 gene | X | 0.699905 | 5.666473 | 3.5136E-08 | 7.3801E-05 |
| *mt-Atp8* | mitochondrially encoded ATP synthase 8 | MT | 70.39237 | 352.9241 | 2.4685E-06 | 0.00274496 |
| *Slc1a2* | solute carrier family 1 (glial high affinity glutamate transporter), member 2 | 2 | 0.956922 | 2.521753 | 4.7759E-06 | 0.00475182 |
| *Mmp13* | matrix metallopeptidase 13 | 9 | 4.184194 | 14.46252 | 8.4455E-06 | 0.00751588 |
| *mt-Nd2* | mitochondrially encoded NADH dehydrogenase 2 | MT | 57.94214 | 281.6958 | 9.1444E-06 | 0.00751588 |
| *mt-Co3* | mitochondrially encoded cytochrome c oxidase III | MT | 142.206 | 711.9994 | 1.2619E-05 | 0.00993988 |
| *mt-Nd3* | mitochondrially encoded NADH dehydrogenase 3 | MT | 42.29412 | 197.4671 | 1.3537E-05 | 0.01010267 |
| *mt-Nd1* | mitochondrially encoded NADH dehydrogenase 1 | MT | 113.662 | 546.6074 | 1.3895E-05 | 0.01010267 |
| *Sh3bgrl* | SH3-binding domain glutamic acid-rich protein like | X | 2.642613 | 7.651401 | 2.081E-05 | 0.0140497 |
| *mt-Atp6* | mitochondrially encoded ATP synthase 6 | MT | 173.4383 | 877.3027 | 2.2358E-05 | 0.0145742 |
| *Gm15247* | predicted gene 15247 | X | 0.262426 | 2.9769 | 2.3861E-05 | 0.01503583 |
| *Cntn1* | contactin 1 | 15 | 0.696668 | 1.804019 | 2.5741E-05 | 0.01550346 |
| *mt-Nd4* | mitochondrially encoded NADH dehydrogenase 4 | MT | 98.79618 | 427.4951 | 3.3069E-05 | 0.01838629 |
| *Gpm6a* | glycoprotein m6a | 8 | 1.300444 | 4.003101 | 3.5897E-05 | 0.0193887 |
| *mt-Cytb* | mitochondrially encoded cytochrome b | MT | 112.7958 | 517.9571 | 4.7909E-05 | 0.02383328 |
| *Snap25* | synaptosomal-associated protein 25 | 2 | 3.27933 | 7.705294 | 5.3662E-05 | 0.02547892 |
| *Tmod2* | tropomodulin 2 | 9 | 0.985529 | 2.216837 | 5.7753E-05 | 0.02627604 |
| *Clk1* | CDC-like kinase 1 | 1 | 2.960835 | 7.16193 | 6.1955E-05 | 0.02723708 |
| *mt-Nd6* | mitochondrially encoded NADH dehydrogenase 6 | MT | 137.2348 | 401.5598 | 7.4347E-05 | 0.03123244 |
| *Pcmtd1* | protein-L-isoaspartate (D-aspartate) O-methyltransferase domain containing 1 | 1 | 1.63118 | 3.903377 | 7.9055E-05 | 0.03248819 |
| *Nexn* | nexilin | 3 | 0.907157 | 2.73911 | 8.4395E-05 | 0.03323747 |
| *mt-Co1* | mitochondrially encoded cytochrome c oxidase I | MT | 304.2912 | 1202.818 | 9.1394E-05 | 0.03499673 |

sTable 2. 23 down-regulated genes in the cochleae of Fabp7 knockout mice.

| Gene name | Description | Chromosome | RPKM | | P-value | FDR |
|---|---|---|---|---|---|---|
| | | | WT | KO | | |
| *Snord3a* | small nucleolar RNA, C/D box 3A | 10 | 60.64163 | 0.120856 | 1.11146E-19 | 2.1011E-15 |
| *Snord3b1* | small nucleolar RNA, C/D box 3B1 | 11 | 204.5929 | 64.7285 | 1.02719E-12 | 9.70896E-09 |
| *Snord3b4_2* | small nucleolar RNA, C/D box 3B4 | 11 | 194.1219 | 65.91439 | 7.07647E-12 | 4.45912E-08 |
| *Snord3b3* | small nucleolar RNA, C/D box 3B3 | 11 | 195.0936 | 65.16871 | 3.20268E-11 | 1.51359E-07 |
| *Snord3b2* | small nucleolar RNA, C/D box 3B2 | 11 | 190.8104 | 67.18328 | 1.10434E-10 | 4.1753E-07 |
| *Gm15564* | predicted gene 15564 | 16 | 66.97385 | 21.2033 | 2.57661E-09 | 6.95832E-06 |
| *B930094E09Rik* | RIKEN cDNA B930094E09 gene | 18 | 4.149664 | 0.759337 | 2.04141E-07 | 0.000385909 |
| *Gm25890* | predicted gene, 25890 | 3 | 103.6947 | 39.96801 | 2.39389E-07 | 0.0004114 |
| *Lars2* | leucyl-tRNA synthetase, mitochondrial | 9 | 115.3335 | 35.25956 | 2.8677E-07 | 0.000451758 |
| *Gm11973* | predicted gene 11973 | 11 | 2.640611 | 0.146863 | 6.24579E-07 | 0.000908235 |
| *Mir6236* | microRNA 6236 | 9 | 3301.069 | 1021.061 | 9.92332E-07 | 0.001339931 |
| *5_8S_rRNA_1* | predicted gene, 55073 | 6 | 38.62397 | 8.48783 | 1.26547E-06 | 0.001594829 |
| *Vaultrc5* | vault RNA component 5 | 18 | 23.4724 | 4.401893 | 1.8485E-06 | 0.002184008 |
| *Rn18s-rs5* | 18s RNA, related sequence 5 | 17 | 9313.823 | 2884.598 | 3.89365E-06 | 0.004089197 |
| *Snord17* | small nucleolar RNA, C/D box 17 | 2 | 662.1657 | 265.1592 | 7.36348E-06 | 0.006959962 |
| *Efhd1os* | EF hand domain containing 1, opposite strand | 1 | 3.32653 | 0.158646 | 1.78312E-05 | 0.012484481 |
| *Rnu1b6* | U1b6 small nuclear RNA | 3 | 68.67319 | 24.41333 | 2.62437E-05 | 0.015503457 |
| *Snord22* | small nucleolar RNA, C/D box 22 | 19 | 52.92287 | 20.89378 | 4.76219E-05 | 0.023833275 |
| *Snord15b* | small nucleolar RNA, C/D box 15B | 7 | 79.937 | 29.28769 | 5.83788E-05 | 0.026276038 |
| *5_8S_rRNA_2* | predicted gene, 54867 | 18 | 51.85413 | 13.75319 | 6.56629E-05 | 0.028211164 |
| *Gm14493* | predicted gene 14493 | X | 5.36747 | 0 | 8.20641E-05 | 0.033007224 |
| *Rnu12* | RNA U12, small nuclear | 15 | 36.91076 | 14.72832 | 9.25644E-05 | 0.034996733 |
| *Gm16867* | predicted gene, 16867 | 14 | 11.44702 | 7.052612 | 9.94152E-05 | 0.036849891 |

sTable 3. 47 increased metabolites in the cochleae of Fabp7 knockout mice.

| Name | Ontology | HMDB | WT | KO | P-value |
|---|---|---|---|---|---|
| Indole-3-carboxylic acid | Indolecarboxylic acids and derivatives | HMDB0003320 | 456.5 | 1060.3 | 5.48598E-10 |
| Maltotetraose | Oligosaccharides | HMDB0001296 | 1082.7 | 2082.9 | 2.59181E-05 |
| Cyclic AMP | 3',5'-cyclic purine nucleotides | HMDB0000058 | 602.7 | 1088.3 | 3.26209E-05 |
| Adenosine 2',3'-cyclic phosphate | 2',3'-cyclic purine nucleotides | HMDB0011616 | 1321.7 | 2247.3 | 8.61176E-05 |
| Cytosine | Pyrimidones | HMDB0000630 | 3120.5 | 5207.3 | 0.000115746 |
| Ornithine | L-alpha-amino acids | HMDB0000214 | 900.5 | 1162.1 | 0.001445799 |
| Sinigrin | Alkylglucosinolates | HMDB0034070 | 598.8 | 985.2 | 0.002591794 |
| Cefditoren | Cephalosporins | HMDB0015199 | 94.5 | 594.1 | 0.003560716 |
| Caffeine | Xanthines | HMDB0001847 | 370.0 | 763.1 | 0.004183338 |
| 2,3-Diaminopropionic acid | L-alpha-amino acids | HMDB0002006 | 28336.8 | 32670.6 | 0.006185408 |
| Phosphorylcholine | Phosphocholines | HMDB0001565 | 611.5 | 915.6 | 0.006868772 |
| Gossypetin | Flavonols | NA | 724.7 | 1006.0 | 0.007209818 |
| Anserine | Hybrid peptides | HMDB0000194 | 3343.2 | 4129.1 | 0.007541355 |
| Uridine triphosphate | Pyrimidine ribonucleoside triphosphates | HMDB0000285 | 278.3 | 732.6 | 0.007630823 |
| Spiramycin | Aminoglycosides | NA | 546.5 | 936.4 | 0.007982675 |
| Aspartylphenylalanine | Dipeptides | HMDB0000706 | 8430.3 | 9666.5 | 0.010105297 |
| Adenosine diphosphate ribose | Purine nucleotide sugars | HMDB0001178 | 228.7 | 2165.9 | 0.010726067 |
| Benzyl methyl sulfide | Secondary carboxylic acid amides | HMDB0031314 | 569.1 | 860.6 | 0.011679383 |
| Glycerophosphocholine | Glycerophosphocholines | HMDB0000086 | 58516.3 | 82788.5 | 0.012271925 |
| Phenylacetaldehyde | Phenylacetaldehydes | HMDB0006236 | 15248.5 | 17202.7 | 0.012559428 |
| Uridine diphosphategalactose | Pyrimidine nucleotide sugars | HMDB0000302 | 298.4 | 2513.7 | 0.012671659 |
| Adipic acid | Medium-chain fatty acids | HMDB0000448 | 2822.8 | 3939.5 | 0.014061876 |
| Vanillin | Methoxyphenols | HMDB0012308 | 937.3 | 1329.0 | 0.01485571 |
| L-Carnitine | Carnitines | HMDB0000062 | 1977.3 | 2340.9 | 0.014914577 |
| 4a-Carbinolamine tetrahydrobiopterin | Phloroglucinols and derivatives | HMDB0002215 | 2186.7 | 2483.5 | 0.015691144 |
| Pantothenic acid | Secondary alcohols | HMDB0000210 | 17626.1 | 20951.5 | 0.018774985 |
| Pipernonaline | Benzodioxoles | HMDB0030339 | 386.7 | 645.7 | 0.019704534 |
| Uridine diphosphate-N-acetylglucosamine | Pyrimidine nucleotide sugars | HMDB0000290 | 898.4 | 3965.3 | 0.019976183 |
| Ginkgolide B | Ginkgolides and bilobalides | HMDB0036861 | 4533.5 | 6901.2 | 0.020764683 |

| | | | | | |
|---|---|---|---|---|---|
| Beta-Glycerophosphoric acid | Glycerophosphates | HMDB0002520 | 3092.7 | 4562.7 | 0.022345319 |
| O-Phosphoethanolamine | Phosphoethanolamines | HMDB0000224 | 1053.7 | 1344.9 | 0.022627335 |
| Daidzin | Isoflavonoid O-glycosides | HMDB0033991 | 1172.3 | 1882.3 | 0.026873687 |
| Acetaminophen | 1-hydroxy-2-unsubstituted benzenoids | HMDB0001859 | 1205.2 | 1553.9 | 0.027391473 |
| Diethylphosphate | Dialkyl phosphates | HMDB0012209 | 652.8 | 929.3 | 0.028819375 |
| Erythritol | Sugar alcohols | HMDB0002994 | 1142.6 | 1298.4 | 0.031678083 |
| Morpholine | Morpholines | HMDB0031581 | 895.8 | 1045.4 | 0.033333452 |
| Cinnamic acid | Cinnamic acids | HMDB0000567 | 3157.9 | 3402.5 | 0.034698625 |
| Rhein | Anthracenecarboxylic acids | HMDB0032876 | 162.7 | 713.1 | 0.034880803 |
| Pilocarpine | Gamma butyrolactones | HMDB0015217 | 1512.1 | 1793.6 | 0.036611529 |
| Pseudohypericin | Benzopyrenes | NA | 47.7 | 239.3 | 0.038986859 |
| Aesculetin | 6,7-dihydroxycoumarins | HMDB0030819 | 428.1 | 1575.4 | 0.039803134 |
| Glycitin | Isoflavonoid O-glycosides | HMDB0002219 | 323.7 | 751.5 | 0.039980432 |
| N-Alpha-acetyllysine | N-acyl-alpha amino acids | HMDB0000446 | 721.7 | 859.1 | 0.040125617 |
| N2-(D-1-Carboxyethyl)-L-arginine | Arginine and derivatives | METPA0471 | 90.3 | 387.9 | 0.043598132 |
| Allantoin | Imidazoles | HMDB0000462 | 232.3 | 568.8 | 0.045363133 |
| Corilagin | Hydrolyzable tannins | HMDB0031457 | 827.8 | 1192.6 | 0.046223423 |
| NAD | (5'->5')-dinucleotides | HMDB0000902 | 231.2 | 525.1 | 0.049729637 |

sTable 4. 67 decreased metabolites in the cochleae of Fabp7 knockout mice.

| Name | Ontology | HMDB | WT | KO | P-value |
|---|---|---|---|---|---|
| Itaconic acid | Branched fatty acids | HMDB0002092 | 6748.2 | 5103.3 | 0.000248099 |
| gamma-Glutamylglutamic acid | Dipeptides | HMDB0011737 | 1893.9 | 786.3 | 0.000513232 |
| Glutamylglutamine | Dipeptides | HMDB0028817 | 2106.4 | 936.1 | 0.001010142 |
| 3-Aminoisobutanoic acid | Beta amino acids and derivatives | HMDB0003911 | 4016.7 | 2945.0 | 0.001263464 |
| 3-Hydroxyvaleric acid | Hydroxy fatty acids | HMDB0000531 | 1836.2 | 1177.1 | 0.001761695 |
| Phenylacetic acid | Benzene and substituted derivatives | HMDB0000209 | 5654.2 | 4340.6 | 0.002 |
| L-Glutamic acid | Glutamic acid and derivatives | HMDB0000148 | 14885.6 | 11246.7 | 0.002220646 |
| Oxoglutaric acid | Gamma-keto acids and derivatives | HMDB0000208 | 873.7 | 570.3 | 0.002430534 |
| Cholesterol | Cholesterols and derivatives | HMDB0000067 | 1386.9 | 845.7 | 0.002665046 |
| Biliverdin | Bilirubins | HMDB0001008 | 1724.3 | 740.8 | 0.002933964 |
| Pyrrolidonecarboxylic acid | Alpha amino acids and derivatives | HMDB0000805 | 8350.7 | 6277.5 | 0.004285932 |
| Xanthosine | Purine nucleosides | HMDB0000299 | 2802.3 | 2308.4 | 0.005 |
| Diacetyl | Alpha-diketones | HMDB0003407 | 705.9 | 345.5 | 0.005808594 |
| Acetic acid | Carboxylic acids | HMDB0000042 | 1352.2 | 998.9 | 0.007798874 |
| Adenine | 6-aminopurines | HMDB0000034 | 1365.1 | 777.0 | 0.007814873 |
| L-Aspartic acid | Aspartic acid and derivatives | HMDB0000191 | 3499.5 | 1936.1 | 0.007982343 |
| Caproic acid | Medium-chain fatty acids | HMDB0000535 | 1615.2 | 973.5 | 0.00858766 |
| Citrinin | Benzopyrans | HMDB0041857 | 2545.1 | 1758.5 | 0.008963996 |
| Creatinine | Alpha amino acids and derivatives | HMDB0000562 | 643.1 | 393.6 | 0.009811242 |
| Ononin | Isoflavonoid O-glycosides | HMDB0033987 | 449.8 | 227.5 | 0.011 |
| L-Alanine | Alanine and derivatives | HMDB0000161 | 3928.9 | 2244.3 | 0.010786012 |
| Propionylglycine | N-acyl-alpha amino acids | HMDB0000783 | 7009.1 | 4114.0 | 0.010792112 |
| L-Palmitoylcarnitine | Acyl carnitines | HMDB0000222 | 12402.5 | 8293.9 | 0.011 |
| PC(16:0/18:1(9Z)) | Phosphatidylcholines | HMDB0007972 | 700772.7 | 431433.2 | 0.012 |
| Rosmarinic acid | Coumaric acids and derivatives | HMDB0003572 | 8510.1 | 4732.0 | 0.012 |
| Chrysin | Flavones | HMDB0036619 | 860.1 | 463.5 | 0.012601859 |
| Maleamate | Straight chain fatty acids | METPA0190 | 3404.3 | 2027.9 | 0.013329133 |
| N-Acetyl-L-aspartic acid | Aspartic acid and derivatives | HMDB0000812 | 31577.0 | 19196.9 | 0.01395486 |
| forskolin | Triterpenoids | NA | 10316.2 | 4885.1 | 0.014543474 |

| | | | | | |
|---|---|---|---|---|---|
| Kanamycin | 4,6-disubstituted 2-deoxystreptamines | HMDB0015303 | 1194.3 | 939.1 | 0.014871435 |
| Dihydromethysticin | Kavalactones | HMDB0030791 | 950.1 | 504.5 | 0.014977175 |
| Mesna | Organosulfonic acids | HMDB0003745 | 3096.1 | 2842.7 | 0.018220652 |
| L-Dopa | Tyrosine and derivatives | HMDB0000181 | 1949.3 | 1037.3 | 0.019044678 |
| Azithromycin | Aminoglycosides | HMDB0014352 | 4290.8 | 2353.0 | 0.019412206 |
| Ethyl tetradecanoate | Fatty acid esters | HMDB0034153 | 3494.3 | 2006.7 | 0.020446375 |
| L-prolyl-L-phenylalanine | Dipeptides | HMDB11179 | 627.5 | 346.8 | 0.021 |
| Phenazopyridine | Aminopyridines and derivatives | HMDB0015506 | 898.0 | 672.4 | 0.021 |
| Citramalic acid | Hydroxy fatty acids | HMDB0000426 | 2167.2 | 1753.3 | 0.021714868 |
| Fosfomycin | Organic phosphonic acids | HMDB0014966 | 656.8 | 355.9 | 0.023 |
| Uridine | Pyrimidine nucleosides | HMDB0000296 | 1021.7 | 520.7 | 0.023 |
| Aminophenazone | Phenylpyrazoles | HMDB0015493 | 21087.3 | 15247.1 | 0.023357729 |
| Urocanic acid | Imidazolyl carboxylic acids and derivatives | HMDB0000301 | 1134.5 | 696.9 | 0.025 |
| Arachidonic acid | Long-chain fatty acids | HMDB0001043 | 22689.3 | 14432.6 | 0.026088172 |
| Piperidine | Piperidines | HMDB0034301 | 789.2 | 482.7 | 0.027 |
| Capric acid | Cholesteryl esters | HMDB0000511 | 2423.4 | 1897.3 | 0.027619702 |
| Citrulline | L-alpha-amino acids | HMDB0000904 | 44472.7 | 25814.3 | 0.028790653 |
| Cefditoren | Cephalosporins | HMDB0015199 | 2964.6 | 2718.3 | 0.02889358 |
| Resolvin D1 | Very long-chain fatty acids | HMDB0003733 | 933.8 | 339.2 | 0.029 |
| 4-Hydroxycinnamic acid | Hydroxycinnamic acids | HMDB0002035 | 13069.8 | 9126.7 | 0.029441709 |
| Creatine | Alpha amino acids and derivatives | HMDB0000064 | 822.7 | 422.2 | 0.030033086 |
| 2-Phosphoglyceric acid | Sugar acids and derivatives | HMDB0000362 | 110562.3 | 72890.1 | 0.030968638 |
| p-Aminobenzoic acid | Aminobenzoic acids | HMDB0001392 | 3017.5 | 2419.8 | 0.031746238 |
| Fumaric acid | Dicarboxylic acids and derivatives | HMDB0000134 | 3951.9 | 2305.8 | 0.032103313 |
| Lithocholic acid | Monohydroxy bile acids, alcohols and derivatives | HMDB0000761 | 5414.5 | 3030.0 | 0.033372398 |
| Paromomycin | 4,5-disubstituted 2-deoxystreptamines | HMDB0015490 | 1975.9 | 1391.7 | 0.034 |
| Stearic acid | Long-chain fatty acids | HMDB0000827 | 2233.3 | 1329.3 | 0.034 |
| Hypotaurine | Sulfinic acids | HMDB0000965 | 3534.4 | 3106.2 | 0.03440665 |
| 11-Eicosenoic acid | Long-chain fatty acids | HMDB0034296 | 1344.5 | 604.7 | 0.034707286 |
| Kaur-16-en-18-oic acid | Kaurane diterpenoids | METPA1653 | 2847.7 | 2058.1 | 0.034748919 |
| LysoPC(16:0) | 1-acyl-sn-glycero-3-phosphocholines | HMDB0010382 | 76537.9 | 39559.8 | 0.035 |

| | | | | | |
|---|---|---|---|---|---|
| Oxypurinol | Xanthines | HMDB0000786 | 46838.1 | 41930.3 | 0.036 |
| 4-Nitrophenol | Nitrophenols | HMDB0001232 | 1138.7 | 635.2 | 0.035730013 |
| L-Phenylalanine | Phenylalanine and derivatives | HMDB0000159 | 7886.7 | 7516.3 | 0.036 |
| 2,6-Dimethoxy-1,4-benzoquinone | P-benzoquinones | HMDB0029673 | 1686.6 | 1285.3 | 0.037594862 |
| LysoPC(0:0/18:0) | 2-acyl-sn-glycero-3-phosphocholines | HMDB0011128 | 52490.3 | 27062.4 | 0.039587925 |
| Heptadecanoic acid | Long-chain fatty acids | HMDB0002259 | 981.1 | 542.0 | 0.042222877 |
| L-Cysteine | L-cysteine-S-conjugates | HMDB0000574 | 372.7 | 148.3 | 0.046405441 |

Supplementary Fig. 1

```
                                 753A
Cdh23_PCR_frag_ 121 TCTCCTCCAGTGAGCCCCGCCCCCAG
WT0              92 TCTCCTCCAGTGAGCCCCGCCCCCAG
WT1              90 TCTCCTCCAGTGAGCCCCGCCCCCAG
WT2              91 TCTCCTCCAGTGAGCCCCGCCCCCAG
WT3              90 TCTCCTCCAGTGAGCCCCGCCCCCAG
WT5              90 TCTCCTCCAGTGAGCCCCGCCCCCAG
KO10             92 TCTCCTCCAGTGAGCCCCGCCCCCAG
KO11             90 TCTCCTCCAGTGAGCCCCGCCCCCAG
KO12             89 TCTCCTCCAGTGAGCCCCGCCCCCAG
KO13             90 TCTCCTCCAGTGAGCCCCGCCCCCAG
KO15             91 TCTCCTCCAGTGAGCCCCGCCCCCAG
                    **************************
```
